# Machine learning for image-based detection of patients with obstructive sleep apnea: an exploratory study

**DOI:** 10.1007/s11325-021-02301-7

**Published:** 2021-02-08

**Authors:** Satoru Tsuiki, Takuya Nagaoka, Tatsuya Fukuda, Yuki Sakamoto, Fernanda R. Almeida, Hideaki Nakayama, Yuichi Inoue, Hiroki Enno

**Affiliations:** 1 Institute of Neuropsychiatry, 91, Bentencho, Shinjuku-ku, Tokyo, 162-0851 Japan; 2 Yoyogi Sleep Disorder Center, Tokyo, Japan; 3 Aging and Geriatric Dentistry, Tohoku University Graduate School of Dentistry, Sendai, Japan; 4 Department of Oral Health Sciences, Faculty of Dentistry, The University of British Columbia, Vancouver, Canada; 5 Rist Inc., Kyoto, Japan; 6 Research Institute for Sustainable Humanosphere, Kyoto University, Kyoto, Japan; 7 Department of Somnology, Tokyo Medical University, Tokyo, Japan; 8 Plasma Inc., Tokyo, Japan

**Keywords:** Obstructive sleep apnea, Oropharyngeal crowding, Artificial intelligence, Machine learning

## Abstract

**Purpose:**

In 2-dimensional lateral cephalometric radiographs, patients with severe obstructive sleep apnea (OSA) exhibit a more crowded oropharynx in comparison with non-OSA. We tested the hypothesis that machine learning, an application of artificial intelligence (AI), could be used to detect patients with severe OSA based on 2-dimensional images.

**Methods:**

A deep convolutional neural network was developed (*n* = 1258; 90%) and tested (*n* = 131; 10%) using data from 1389 (100%) lateral cephalometric radiographs obtained from individuals diagnosed with severe OSA (*n* = 867; apnea hypopnea index > 30 events/h sleep) or non-OSA (*n* = 522; apnea hypopnea index < 5 events/h sleep) at a single center for sleep disorders. Three kinds of data sets were prepared by changing the area of interest using a single image: the original image without any modification (full image), an image containing a facial profile, upper airway, and craniofacial soft/hard tissues (main region), and an image containing part of the occipital region (head only). A radiologist also performed a conventional manual cephalometric analysis of the full image for comparison.

**Results:**

The sensitivity/specificity was 0.87/0.82 for full image, 0.88/0.75 for main region, 0.71/0.63 for head only, and 0.54/0.80 for the manual analysis. The area under the receiver-operating characteristic curve was the highest for main region 0.92, for full image 0.89, for head only 0.70, and for manual cephalometric analysis 0.75.

**Conclusions:**

A deep convolutional neural network identified individuals with severe OSA with high accuracy. Future research on this concept using AI and images can be further encouraged when discussing triage of OSA.

**Supplementary Information:**

The online version contains supplementary material available at 10.1007/s11325-021-02301-7.

## Introduction

Various degrees of craniofacial impairment, including a large tongue relative to the maxilla and mandible (i.e., oropharyngeal crowding) and caudal displacement of the hyoid bone (i.e., a low hyoid), are involved in the presence/development of most cases of obstructive sleep apnea (OSA) [1–7]. Although focusing on this phenotypic feature in 2-dimensional lateral cephalometric radiographs is not new in itself [1, 3–6], it is still an appealing approach for the detection of OSA because the use of images is simple and can help prevent human subjectivity from influencing the diagnostic process, as compared with questionnaires [8].

Machine learning, an application of artificial intelligence (AI), is the study and development of systems that can learn from and make predictions about data without the need to be programmed [9–12]. If the ground truth of every cephalometric image used for the training/testing model is labeled based on an OSA/non-OSA diagnosis by polysomnography, such images may become an optimal target of machine learning. Considering that patients with severe OSA exhibit a more crowded oropharynx [2, 5], it would be reasonable to test the hypothesis that a machine learning model could be used to differentiate severe OSA and non-OSA by 2-dimensional images: two different populations regarding craniofacial morphology. The clinical implications of the findings will be discussed later.

## Methods

### Study participants

**Fig. 1:**
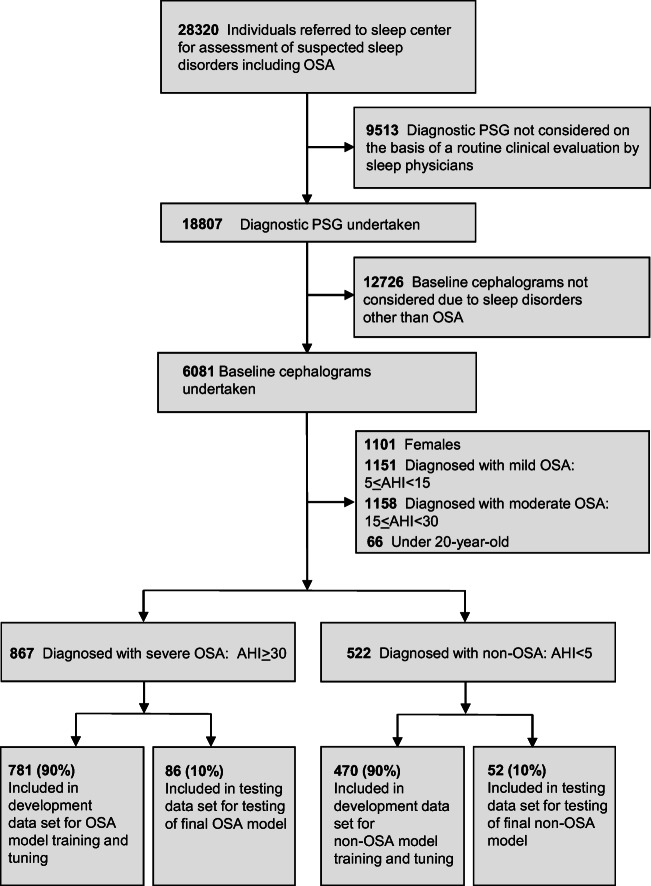
Data sets for the development and testing of a deep convolutional neural network. AHI, apnea hypopnea index; OSA, obstructive sleep apnea; PSG, polysomnography

The present study was conducted in accordance with the amended Declaration of Helsinki and followed the Transparent Reporting of a multivariable Prediction Model for Individual Prognosis or Diagnosis (TRIPOD) reporting guidelines [13]. The study was designed and performed at the Yoyogi Sleep Disorder Center (Tokyo, Japan) and the study protocol was approved by the Ethics Committee of the Institute of Neuropsychiatry, Tokyo, Japan (approval no. 176). Individuals who had undergone diagnostic polysomnography from March 2006 to February 2017 provided their written informed consent for the anonymous use of their data, including polysomnography, laboratory values, and information on images (*n* = 18,807) (Fig. 1). Every patient who was suspected to have OSA was diagnosed with either OSA or non-OSA based on initial diagnostic polysomnography according to standard procedures [14]. All of these patients underwent lateral cephalometric radiography with the use of one identical device for the evaluation of craniofacial and upper airway structure in the time frame of 2006 to 2017 (*n* = 6081). The severity of OSA was assessed in terms of AHI (mild [AHI ≥ 5 – < 15 events/h sleep], moderate [AHI ≥ 15 – < 30 events/h sleep], or severe [AHI ≥ 30 events/h sleep]) while subjects with AHI less than 5 were assumed to be non-OSA [14]. We included male subjects in whom AHI was more than and/or equal to 30 events/h sleep (patient group) or less than 5 events/h sleep (controls). Exclusion criteria included females (*n* = 1101) and mild (*n* = 1151) to moderate (*n* = 1158) OSA patients. We also excluded patients under 20 years of age due to the possibility of ongoing growth and development of craniofacial bony tissues (*n* = 66). Consequently, a total of 1389 consecutive patients who met the inclusion/exclusion criteria were divided into 2 groups: OSA patients (*n* = 867) and controls (*n* = 522).

### Deep convolutional neural network model

**Fig. 2:**
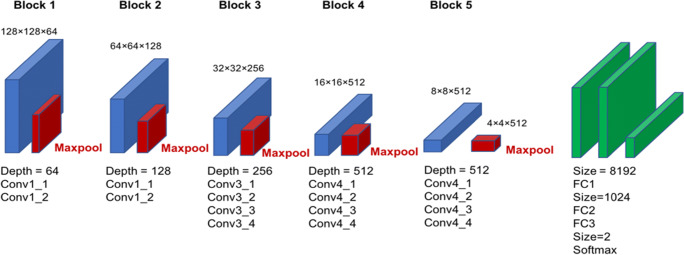
Overall architecture of a deep convolutional neural network model for detection of obstructive sleep apnea. Conv, convolutional layer; FC, fully connected layer; Maxpool, maximum pooling layer

A deep convolutional neural network (DCNN) model called the Visual Geometry Group (VGG-19), which is a deep learning architecture that is part of a broader family of machine learning methods in AI technology, was used (Fig. 2). This type of DCNN is configured to automatically learn local features of images and generate a classification model [15, 16]. The aspect ratio of the original images was 2010 × 1670 pixels; however, for the analysis, we changed the aspect ratio of all input images and resized them to 128 × 128 pixels. As the red-green-blue input of images had a range of 0–255, it was first normalized to a range of 0–1 by dividing by 255.

The VGG-19 model is comprised of five blocks and three fully connected layers. Each block includes convolutional layers followed by a max pooling layer with decreasing position sensitivity but greater generic recognition. Flattening of the output from block 5 results in only three fully connected layers. The first layer removes spatial information from the extracted feature vectors, and the second layer is a classification layer that uses feature vectors from target images acquired in previous layers in combination with the softmax function and binary classification. To improve generalization performance, dropout processing was performed such that masking was achieved with a probability of 50% in the first fully connected layer. Fine tuning was used to increase the learning speed and achieve higher performance with less data. We used the following parameters from ImageNet: blocks 1 to 5 were fixed, whereas the fully connected layers were trained. The weights of the fully connected layers were optimized using a stochastic gradient descent algorithm with momentum (learning coefficient = 0.00001, decay = 1e-6, momentum = 0.9).

Prior to the input of images to the model at each epoch, augmented processing of images with regard to rotation angle, horizontal shift, vertical shift, and horizontal reversal was randomly performed to obtain a robust model. Learning was carried out with mini-batch processing of 2 images and an epoch number of 1000. During the learning phase, we saved the models after 100, 400, 700, and 1000 epochs. After learning, we selected the model with the highest accuracy for test data among these four deep learning models. For this purpose, Keras (https://keras.io/ja/) was run on TensorFlow (https://www.tensorflow.org/) written in Python and was used to build and evaluate the model. We trained the model using a Core™ i7-7700K CPU (Intel) and a GeForce GTX 1080 Ti GPU (NVIDIA).

### Image dataset

**Fig. 3:**
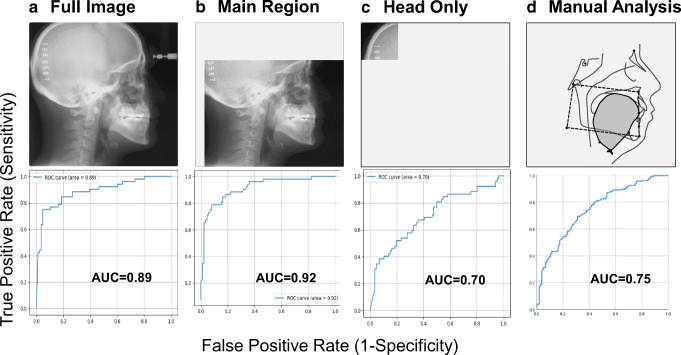
Image data sets (*upper*) and area under the receiver-operating characteristic (ROC) curve for detection of obstructive sleep apnea (*lower*). AUC, area under the curve. *Note* that the ROC curve with the better AUC (i.e., 0.75) obtained by a less crowded oropharynx and hyoid position is shown as the representative result of manual cephalometric analyses (Table 3)

Every cephalometric image was taken of the natural head posture determined by visual feedback in a mirror in accordance with the established method [17]. Among the total 1389 images, 1251 (90%) images were used for training (i.e., learning images; 781 OSA images, and 470 non-OSA images) and the remaining 138 (10%) images were used for testing (i.e., testing images, 86 OSA images, and 52 control images) in accordance with recent reports [18–21]. To investigate which part of the original image is focused upon when using DCNN, three data sets were prepared by changing the area of interest (Fig. 3). One set included full images without any modification (full image; Fig. 3A upper). Another set included images of the area of interest to which skilled sleep-related personnel often pay particular attention in discussing the likelihood of OSA (main region; Fig. 3B upper). When the forehead clamp was present in an image, it was detected by a template matching algorithm (matching rate 0.8 or more). Cropping of the image was then executed by specifying the area with reference to the detected coordinates. Consequently, the main region includes the facial profile, upper airway, and craniofacial soft/hard tissues such as the tongue, soft palate, maxilla, mandible, teeth, and cervical bones from the lower right corner of the original image. The other set included images in which part of the occipital region (head only; 400 × 400 pixels from the upper left corner of the original image) was picked up for comparison with the outcomes with the full image and main region (Fig. 3C upper). For comparison with the results of the DCNN analyses, cephalometric parameters were also measured manually by a radiologist (YT) and an orthodontist (ST) as described in our previous reports without knowledge of the OSA severity of each participant (Fig. 3D upper) [3, 5]. Detailed information for this manual cephalometric measurements and the analysis of intra-rater reliability were as described previously (Online resource 1 and 2). The repeated assessment of manual cephalometric measurements on different days (day 1 vs day 2) yielded good reproducibility, with an intraclass correlation coefficient (95% CI) of 0.9970 (0.9951–0.9982) [22].

### Statistical analysis

Data are presented as the mean ± standard deviation and unpaired *t* tests were used to compare each variable between the OSA group and non-OSA group without adjusting for baseline characteristics (SPSS version 25, SPSS Japan). The primary outcome was diagnostic accuracy in terms of area under the curve (AUC) of the receiver-operating characteristic (ROC) curve, both of which were computed with the use of Matplotlib (version 3.0.3) for DCNN analysis (Fig. 3A, B, and C) or SPSS for manual cephalometric analysis (Fig. 3D). When the predictive score obtained from the DCNN analysis exceeded the threshold (i.e., cutoff value = 0.50), it was judged to be positive (i.e., OSA). The DCNN model was fitted to only 90% of the test data while the remaining 10% of the data were thinned out. For the manual cephalometric analysis, the patients were divided into two groups according to the degree of oropharyngeal crowding and further classified into two subgroups based on hyoid position (Online Resource 3) [3, 5]. We prepared a 2 × 2 cross table for the *χ*^*2*^ test and a similar table with two layers to evaluate the effects of the combined use of the two parameters for the detection of OSA. We further compared the predictive quality of the DCNN model to that of manual cephalometric analyses using sensitivity, specificity, positive likelihood ratio, negative likelihood ratio, positive predictive value, negative predictive value, and AUC [23, 24]. A *p* value of < 0.05 was considered to indicate statistical significance.

## Results

There were significant differences in age (*p* < 0.01), AHI (*p* < 0.01), and body mass index (BMI) (*p* < 0.01) between patients with OSA and non-OSA samples (Table 1). After the development of the DCNN model on the basis of 90% of the total images (*n* = 1251), its ability to predict OSA was then tested using the remaining 10% (*n* = 138). The DCNN as well as manual cephalometric analyses significantly predicted the presence of severe OSA: full image (*χ*^*2*^ = 62.5, *P* < 0.01), main region (*χ*^*2*^ = 59.2, *p <* 0.01), head only (*χ*^*2*^ = 12.7, *p* < 0.01), and combinations of a more crowded oropharynx and hyoid position (*χ*^*2*^ = 39.7, *p* < 0.01) and a less crowded oropharynx (*χ*^*2*^ = 31.8, *p* < 0.01) (Table 2 and Online Resource 3).

**Table 1:** Baseline demographics of the two populations

Patient characteristics	OSA	non-OSA
*n* (%)	867	522
Age (years)	49.7 ± 8.9^a^	41.2 ± 13.0
BMI (kg/m^2^)	28.2 ± 5.5 ^a^	23.8 ± 3.7
AHI (events/h sleep)	54.0 ± 20.1 ^a^	2.5 ± 1.4

*AHI* apnea hypopnea index, *BMI* body mass index, *OSA* obstructive sleep apnea. ^a^*p* < 0.01 versus non-OSA

**Table 2:** Detection of obstructive sleep apnea with a deep convolutional neural network and manual cephalometric analysis

True label					
	Predicted		OSA	Non-OSA	Total
DCNN analysis					
	Full image	OSA	77	12	89
		Non-OSA	9	40	49
		Total	86 ^a^	52	138
	Main region	OSA	79	15	94
		Non-OSA	7	37	44
		Total	86 ^b^	52	138
	Head only	OSA	73	30	103
		Non-OSA	13	22	35
		Total	86 ^c^	52	138
Manual cephalometric analysis					
More crowded oropharynx		Low hyoid	241	20	261
		No low hyoid	82	40	122
		Total	323 ^d^	60	383
Less crowded oropharynx		Low hyoid	108	21	129
		No low hyoid	91	82	173
		Total	199 ^e^	103	302

The DCNN analyses were based on 138 test images. ^a^*Χ*^2^ = 62.5, *P* < 0.01 versus non-OSA. ^b^*Χ*^2^ = 59.2, *P* < 0.01 versus non-OSA. ^c^*Χ*^2^ = 12.7, *P* < 0.01 versus non-OSA. ^d^*Χ*^2^ = 39.7, *P* < 0.01 versus non-OSA. ^e^*Χ*^2^ = 31.8, *P* < 0.01 versus non-OSA. *DCNN* deep convolutional neural network, *OSA* obstructive sleep apnea

Table 3 shows the predictive qualities of the DCNN model and manual cephalometric analyses. The sensitivity/specificity and positive likelihood ratio/negative likelihood ratio were 0.90/0.77 and 3.88/0.14 for full image, 0.84/0.81 and 4.35/0.20 for main region, and 0.71/0.63 and 1.91/0.46 for head only, respectively. Positive predictive value and negative predictive value were 0.87/0.82 in the full image group and 0.88/0.75 and 0.85/0.42 in the main region and head only groups, respectively. Test loss in the main region group (1.16) was less than those in the full image (1.35) and head only groups (3.08), while the full image group had the highest test accuracy (0.85) of the three categories for DCNN analyses (0.83 for main region and 0.69 for head only). Similarly, the detailed results from the manual cephalometric analysis are shown in Online Resource 3 and Table 3. Manual cephalometric analyses demonstrated more oropharyngeal crowding in terms of TG/LFC (*p* < 0.01) and a lower hyoid with reference to MP-H (*p* < 0.01) in the OSA group in comparison with the non-OSA group (Online Resource 3). The sensitivity/specificity and positive likelihood ratio/negative likelihood ratio for the combination of a more crowded oropharynx and hyoid position were 0.75/0.67 and 2.24/0.38, respectively (Table 3). The sensitivity/specificity and positive likelihood ratio/negative likelihood ratio for the combination of a less crowded oropharynx and hyoid position were 0.54/0.80 and 2.66/0.57, respectively. Higher positive predictive values were observed for a more crowded oropharynx and hyoid position (0.92) and for the combination of a less crowded oropharynx and hyoid position (0.84), whereas negative predictive values for these combinations were 0.33 and 0.47, respectively. The AUC in the main region group (0.92) was higher than those in the full image (0.89) and head only (0.70) groups, while those in the more crowded oropharynx group and less crowded oropharynx group were 0.73 and 0.75, respectively (Table 3 and Fig. 3). Accordingly, the AUCs obtained in the full image and main region groups using DCNN outperformed those from the manual cephalometric analysis.

**Table 3:** Comparison of predictive qualities of the deep convolutional neural network model to that of manual cephalometric analysis

	DCNN analysis			Manual cephalometric analysis	
	Full image	Main region	Head only	More crowded oropharynx and hyoid position	Less crowded oropharynx and hyoid position
Sensitivity	0.90	0.84	0.71	0.75	0.54
Specificity	0.77	0.81	0.63	0.67	0.80
LR+	3.88	4.35	1.91	2.24	2.66
LR-	0.14	0.20	0.46	0.38	0.57
PPV	0.87	0.88	0.85	0.92	0.84
NPV	0.82	0.75	0.42	0.33	0.47
AUC	0.89	0.92	0.70	0.73	0.75

The best cutoff values for the hyoid position and oropharyngeal crowding in the manual cephalometric analyses were determined by receiver-operating characteristic curves, respectively (Supplemental Table S1). *AUC* area under the curve, *DCNN* deep convolutional neural network, *LR+* positive likelihood ratio, *LR-* negative likelihood ratio, *NPV* negative predictive value, *PPV* positive predictive value

## Discussion

In this exploratory study, a DCNN identified individuals with severe OSA through the use of 2-dimensional radiographs. Lateral cephalometric radiographs have not been used for the diagnosis of OSA, but have been used to help with the evaluation of craniofacial morphology in OSA patients as well as in subjects with dental malocclusion because the pathogeneses of both OSA and dental malocclusions are closely related to craniofacial soft and hard tissue structures [25]. However, it may be reasonable to use DCNN and lateral cephalometric radiographs for OSA detection considering the recent development of machine learning technologies in parallel with, and their high affinity for, medical images.

Although we succeeded in demonstrating that a DCNN differentiated severe OSA and non-OSA, our current DCNN model does not suggest at all that primary and/or tertiary care settings are now better equipped to identify severe OSA solely by the use of images because of significant limitations. First, as we mentioned in the “Introduction” section, our original purpose was to test whether AI could identify patients with severe OSA who have a more crowded oropharynx than non-OSA individuals [2, 5]. Thus, before we considered developing a model for predicting OSA that could be used in a clinical setting, we exploratorily prepared labeled dichotomized samples as in the standard method for supervised learning in deep learning approaches. As shown in Online Resource 4, we additionally attempted to validate our model using another 269 consecutive male samples with a wide range of OSA severity who visited our center from May 2018 to December 2018. Within this more real-world dataset, labeled patients with mild to moderate OSA (*n* = 148) had to be classified as either severe OSA (*n* = 95) or non-OSA (*n* = 53) by the DCNN diagnosis, since our model did not learn the craniofacial features of mild to moderate OSA. This phenomenon is consistent with the recent speculation that “a machine learning algorithm trained on a clinic sample of predominantly men with mostly severe OSA would likely poorly perform in a population-based dataset of men and women with wide range of OSA severity and subtypes” [12]. On the other hand, it is still notable that the main region model succeeded in detecting 92 (92%) of 100 severe OSA patients, while the full image model found 87 (90%), among the above 269 samples (Online Resource 4 and Online Resource 5). This supplementary data suggests that recognition of a craniofacial structure specific for OSA using 2-dimensional images may be a suitable application of machine learning techniques and that extension of the concept of our study to another study that includes mild to moderate OSA samples may provide a practical model for predicting OSA in the future. Second, subjects were all recruited from a single tertiary sleep center and thus non-OSA subjects differ from community samples, which is a significant limitation of our study and other similar studies overall; it is not feasible to prospectively perform polysomnography and label the ground truth in samples with AHI less than 5 events/h sleep from the general population. Third, the 1389 subjects analyzed in this study might be the biggest data set for a cephalometric OSA study ever. However, the subjects included only males because of both the limited number of female learning/testing data and possible craniofacial differences between sexes [26]. Our samples reflect the usual demographics of sleep clinic populations and, therefore, replication of these findings in a larger set of female samples is necessary. In addition, a neuromuscular compensation mechanism is more augmented in defense of the upper airway in female OSA patients as compared with male OSA individuals [27]. Therefore, female OSA patients could have the more crowded oropharynx if the severity of OSA is the same between male and female samples. This indicates that our DCNN model might detect female OSA more easily: a better AUC than that from male samples could be obtained. Fourth, we included only one ethnic group and confirmation of our results by the inclusion of ethnic groups other than Asian would be interesting and necessary. The authors believe that these significant limitations do not necessarily undermine the concept of the future use of AI and images for objectively detecting patients with OSA.

In the present study, we assumed that the DCNN model achieved the higher accuracy of ROC analyses (i.e., AUC = 0.92 from the main region) when the predictive ability was classified based on AUC (excellent = 0.9 to 1, good = 0.8 to 0.9, fair = 0.7 to 0.8, poor = 0.6 to 0.7, or non-discriminative = 0.5 to 0.6) [23, 24]. The higher AUC from the main region (0.92) relative to those from the full image (0.89) and head only (0.70) may reflect a certain anatomical background that supports our hypothesis; the DCNN, similar to a manual analysis, might also focus more on the oropharyngeal region, which is an area of interest in OSA images for skilled radiologist/sleep-related personnel (Fig. 3) [3, 5]. The higher accuracy of ROC analyses with 2-dimensional images alone using DCNN models may suggest that an anatomical complication is a major cause of OSA among various known/unknown factors in the middle-aged OSA population. In the present study, the impact of different OSA phenotypes other than craniofacial anatomy (e.g., upper airway muscle responsiveness, breathing control, arousability, etc.) on the results is unknown. However, clustering OSA in future studies could contribute to increasing the accuracy of the DCNN analyses. In contrast to good (full image) and excellent (main region) accuracy, the fair accuracy (AUC 0.70) obtained solely from the occipital region (head only) was unexpected, but of interest [23, 24]. Since OSA patients are a significantly older and more obese population than controls (Table 1), and the risk of OSA increases with aging and obesity, the DCNN might recognize information related to age and/or obesity in an image (e.g., loss of bone mineral density, subcutaneous fat thickness), which is not apparent even to experts [28, 29].

A strength of this study was the quality of the training/test data with a large sample size; samples were obtained from a homogenous single cohort with the standard method for taking lateral cephalometric radiographs, and the diagnosis of OSA/non-OSA was achieved by standard nocturnal polysomnography, resulting in adequately labeled ground truth and thereby maximally avoiding annotation noise [12]. Furthermore, there are some clinical implications. Since simplicity, quickness, inexpensiveness, and low-dose radiation support the practical use of 2-dimensional radiographs relative to 3-dimensional images, the concept of the present study could be widely applicable in dental offices as well as primary care settings/satellite practices. Undoubtedly, the patient’s subjective symptoms of OSA (i.e., excessive daytime sleepiness, snoring, etc.) are the simplest signs for the early detection of OSA [8]. However, subjective assessment often introduces noise to the initial diagnostic process and accuracy. The machine learning technique is objective (i.e., no inclusion of human subjectivity in the diagnostic process), less labor-intensive, and less time-constrained and should be able to minimize the delay of both the diagnosis and the referral of patients to secondary/tertiary care. Accordingly, the combination of demographic characteristics including anthropometric features may provide different AI models that maintain the clinical usefulness of a DCNN model in the detection of OSA [30–32].

## Conclusion

A deep convolutional neural network, a deep learning architecture that is part of a broader family of machine learning methods in AI technology, accurately identified individuals with severe OSA using 2-dimensional lateral cephalometric radiographs. Future research on this concept using AI and 2-dimensional images per se can be further encouraged when discussing triage of OSA.

## Supplementary information

ESM 1 (PDF 862 kb)

## Data Availability

Data are available upon request.
