# Supplementary material for: Machine learning for image-based detection of patients with obstructive sleep apnea: an exploratory study
Source: Sleep Breath. 2021 Feb 8;25(4):2297–305. doi: 10.1007/s11325-021-02301-7 (PMC8590647; doi:10.1007/s11325-021-02301-7)
Supplement: Supplementary file 1 — (PDF 862 kb) [file 11325_2021_2301_MOESM1_ESM.pdf]

Supplementary material

Article title: Machine learning for image-based detection of patients with obstructive sleep apnea: an exploratory study

Journal name: Sleep and Breathing

Author names: *Satoru Tsuiki・Takuya Nagaoka・Tatsuya Fukuda・Yuki Sakamoto・Fernanda R. Almeida・Hideaki Nakayama ・ Yuichi Inoue ・ Hiroki Enno

***Affiliation and e-mail address of the corresponding author:**

Satoru Tsuiki, DDS, PhD
Institute of Neuropsychiatry
5-10-10 Yoyogi, Shibuya-ku, Tokyo 151-0053, Japan
E-mail: tsuiki@somnology.com

Supplementary material

Online Resource 1

Manual cephalometric assessment

For the purpose of cephalometric analyses by a radiologist (YT) and an orthodontist (ST), images in which it was difficult to visually identify the silhouettes of soft/hard tissues were robustly excluded (n=704). Consequently, a total of 685 images (522 patients with OSA and 163 controls) were selected for cephalometric assessment. Data analysis was performed from April 1, 2018 to August 31, 2019.

The lower face cage (LFC; dotted trapezoid in Supplementary Figure S1 in Online Resource 2) was determined as the maxillomandibular size (i.e., bony enclosure size of the upper airway). The tongue size (TG) was defined as the area outlined by the dorsal configuration of the tongue surface and lines that connect the tongue tip, retrognathion, hyoid bone, and base of the epiglottis. By using these definitions, oropharyngeal crowding was calculated as the ratio between TG and LFC (i.e., TG/LFC) [S1-S3]. We also measured the linear distance from the mandibular plane to the hyoid bone (MP-H) as a result of excessive soft tissue within the maxillomandibular enclosure [S1-S3]. The analysis of intra-rater reliability for the above manual cephalometric measurements yielded an intraclass correlation coefficient of 0.9970 (95% confident interval: 0.9951–0.9982), as previously reported [S4,S5].

Supplementary references

S1. Tsuiki S, Isono S, Ishikawa T, Yamashiro Y, Tatsumi K, Nishino T. Anatomical balance of the upper

airway and obstructive sleep apnea. *Anesthesiology.* 2008;**108**(6):1009-1015.

S2. Ito E, Tsuiki S, Namba K, Takise Y, Inoue Y. Upper airway anatomical balance contributes to optimal continuous positive airway pressure for Japanese patients with obstructive sleep apnea syndrome. *J Clin Sleep Med.* 2014;**10**(2):137-142.

S3. Ito E, Tsuiki S, Maeda K, Okajima I, Inoue Y. Oropharyngeal crowding closely relates to aggravation of OSA. *Chest.* 2016;**150**(2):346-352.

S4. Maeda K, Tsuiki S, Isono S, Namba K, Kobayashi M, Inoue Y. Difference in dental arch size between obese and non-obese patients with obstructive sleep apnoea. *J Oral Rehabil.* 2012;**39**(2):111-117.

S5. Maeda K, Tsuiki S, Fukuda T, Takise Y, Inoue Y. Is maxillary dental arch constriction common in Japanese male adult patients with obstructive sleep apnoea? *Eur J Orthod*. 2014;**36**(4):403-408.

Supplementary material

Online Resource 2

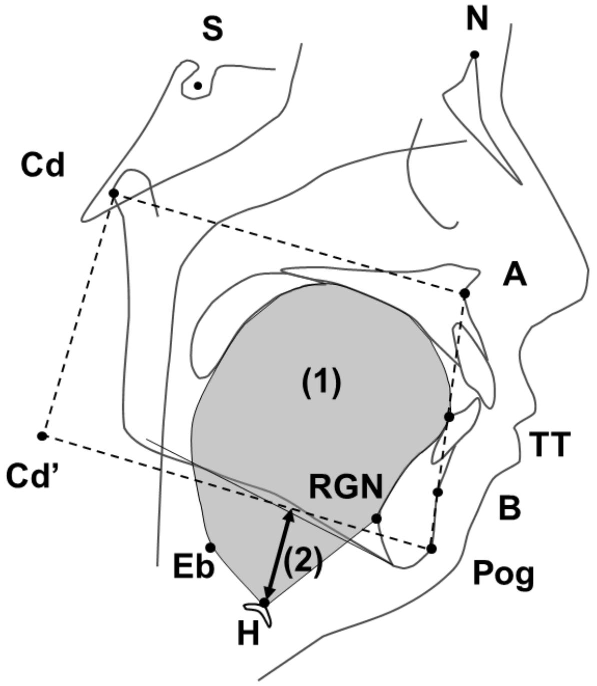

Supplementary Figure S1 Definitions of cephalometric variables for manual analysis [S1-S3].

S=sella; A=subspinale; B=supramentale; Cd=medial condylar point of the mandible; Cd'=the point where Pog projects on the line perpendicular to the Cd-A line at the Cd point; Eb=base of epiglottis; H=hyoid bone; MP=mandibular plane, N=nasion; Pog=pogonion; RGN=retrognathia; TT=tongue tip. (1) Tongue (TG): tongue size is defined as the area outlined by the dorsal configuration of the tongue surface and lines that connect the TT, RGN, H, and Eb. The lower face cage (LFC) is defined as a trapezoid by Cd-A-Pog-Cd' (dotted lines). (2) MP-H: perpendicular distance from the anterosuperior point of the hyoid bone to the mandibular plane.

Supplementary material

Online Resource 3

		OSA	Non-OSA	Total
**A**	**n**	522	163	685
	**TG/LFC**	0.63±0.06[a]	0.60±0.06	-
	**MP-H**	23.7±6.3[b]	17.7±6.1	-
	**SNA**	83.4±3.7	83.1±3.6	-
	**SNB**	79.5±3.9	79.2±4.0	-
**B**		**OSA**	**Non-OSA**	**Total**
**More crowded oropharynx**		323	60	383
**Less crowded oropharynx**		199	103	302
		522[c]	163	685
**Low hyoid**		349	41	390
**No low hyoid**		173	122	295
		522[d]	163	685
**More crowded oropharynx**	**Low hyoid**	241	20	261
	**No low hyoid**	82	40	122
	**Total**	323[e]	60	383
**Less crowded oropharynx**	**Low hyoid**	108	21	129
	**No low hyoid**	91	82	173
	**Total**	199[f]	103	302

Supplementary Table 1 Cephalometric characteristics of the OSA and control groups (A) and use of cut-off values for oropharyngeal crowding and the hyoid position alone or in combination for predicting OSA (B). TG/LFC: tongue size relative to lower face cage, MP-H: distance between mandibular plane to hyoid, OSA: obstructive sleep apnea, SNA: angle between the nasion (N)–sella (S) line and the line from point A (subspinale) to N, SNB: angle between the N–S line and the line from point B (supramentale) to N. More

Supplementary material

crowded = TG/LFC>0.61, Less crowded = TG/LFC<0.61, Low hyoid = MP-H > 20.85 mm, No low hyoid = MP-H $\leq$ 20.85 mm. These best cut-off values for oropharyngeal crowding and hyoid position in this study were determined by a receiver-operating characteristic curve analysis (. [a] P<0.01 versus non-OSA, [b] P<0.01 versus non-OSA, [c] χ^2 =31.7, p<0.01 versus OSA, [d]χ^2 =88.1, p<0.01 versus OSA, [e]: χ^2 =39.7, p<0.01 versus OSA, [f]: χ^2 =31.8, p<0.01 versus OSA.

Supplementary material

Online Resource 4

Deep convolutional neural network analysis

**A**				**True label**		
			**Severe OSA**	**mild to moderate OSA**	**Non-OSA**	**Total**
**Predicted**	**Full Image**	**Severe OSA**	87	95	10	192
		**Non-OSA**	13	53	11	77
		**Total**	100	148	21	269
	**Main region**	**Severe OSA**	92	95	8	195
		**Non-OSA**	8	53	13	74
		**Total**	100	148	21	269
**B**						
**Predicted**	**Full Image**	**Severe OSA**	87	-	10	97
		**Non-OSA**	13	-	11	24
		**Total**	100[a]	-	21	121
	**Main Region**	**Severe OSA**	92	-	8	100
		**Non-OSA**	8[b]	-	13	21
		**Total**	100	-	21	121

Supplementary Table 2 Detection of OSA with a deep convolutional neural network in 269 consecutive male samples with a wide range of OSA severity. Abbreviations are the same as those in Table S1. Deep convolutional neural network classified mild to moderate OSA (n=148) into either severe OSA or non-OSA since the network did not learn images from mild to moderate OSA (A). The model still maintains fair accuracy when excluding mild to moderate OSA (B). [a]χ^2 =16.9, P<0.01 versus non-OSA, [b]χ^2 =35.2, P<0.01 versus non-OSA.

Supplementary material

Online Resource 5

	Deep convolutional neural network analysis	
	**Full Image**	**Main Region**
**Sensitivity**	0.87	0.92
**Specificity**	0.52	0.62
**LR+**	1.83	2.42
**LR-**	0.25	0.13
**PPV**	0.90	0.92
**NPV**	0.46	0.62

Supplementary Table 3 Predictive qualities of the deep convolutional neural network model based on 121 of 269 consecutive samples in Supplementary Table 2B (Online Resource 4). LR+ = positive likelihood ratio; LR- = negative likelihood ratio; NPV = negative predictive value; PPV = positive predictive value.
